# Novel Anti-Tuberculosis Nanodelivery Formulation of Ethambutol with Graphene Oxide

**DOI:** 10.3390/molecules22101560

**Published:** 2017-10-12

**Authors:** Bullo Saifullah, Alina Chrzastek, Arundhati Maitra, Bullo Naeemullah, Sharida Fakurazi, Sanjib Bhakta, Mohd Zobir Hussein

**Affiliations:** 1 Mycobacteria Research Laboratory, Department of Biological Sciences, Institute of Structural and Molecular Biology (ISMB), Birkbeck, University of London, Malet Street, London WC1E 7HX, UK; bullosaif1@gmail.com (B.S.); alina.chrzastek@gmail.com (A.C.); arundhati.mrl@gmail.com (A.M.); s.bhakta@bbk.ac.uk (S.B.); 2 Material Synthesis and Characterization Laboratory, Institute of Advanced Technology (ITMA), Universiti Putra Malaysia (UPM), Serdang 43400, Selangor, Malaysia; 3 Laboratory for Vaccine and Immunotherapeutics, Institute of Biosciences, Universiti Putra Malaysia (UPM), Serdang 43400, Selangor, Malaysia; sharida@upm.edu.my; 4 Department of Neurology (Ward No. 18) Jinnah Postgraduate Medical Center/Jinnah Sindh Medical, University Karachi, Karachi 75510, Pakistan; bullonaeem@hotmail.com; 5 Department of Human Anatomy Faculty of Medicine and Health Sciences, University Putra Malaysia (UPM), Serdang 43400, Selangor, Malaysia

**Keywords:** graphene oxide, nanocarrier, ethambutol, tuberculosis, nanodelivery formulation

## Abstract

Tuberculosis (TB) is a bacterial disease responsible for millions of infections and preventable deaths each year. Its treatment is complicated by patients’ noncompliance due to dosing frequency, lengthy treatment, and adverse side effects associated with current chemotherapy. However, no modifications to the half-a-century old standard chemotherapy have been made based on a nanoformulation strategy to improve pharmacokinetic efficacy. In this study, we have designed a new nanodelivery formulation, using graphene oxide as the nanocarrier, loaded with the anti-TB antibiotic, ethambutol. The designed formulation was characterized using a number of molecular analytical techniques. It was found that sustained release of the drug resulted in better bioavailability. In addition, the designed formulation demonstrated high biocompatibility with mouse fibroblast cells. The anti-TB activity of the nanodelivery formulation was determined using whole-cell resazurin microtiter plate assay, modified-spot culture growth inhibition assay, and biofilm inhibition assay. The nanodelivery formulation showed good anti-mycobacterial activity. The anti-mycobacterial activity of Ethambutol was unaffected by the drug loading and release process. The results of this study demonstrated the potential of this new nanodelivery formulation strategy to be considered for modifying existing chemotherapy to yield more efficacious antibiotic treatment against TB.

## 1. Introduction

Despite leaps in technological advancement, tuberculosis (TB) is still killing 1.5 million people every year. TB treatment is complicated by patient noncompliance due to the side effects of the currently available drugs, frequent dosing, and the lengthy treatment duration [1,2,3]. Nanotechnology is a rapidly advancing scientific field with a wide range of applications in electronics, healthcare, and engineering, that can potentially be used for anti-TB treatment [4,5,6]. In healthcare, nanomedicine for the delivery of therapeutics is receiving attention because of the fascinating properties of nanoparticles that are absent from their bare counterparts [7]. Graphene and its derivative materials are being widely explored and applied in various fields of science and technology, such as electronics, material science, sensing technology, engineering photonics, and biomedical sciences [8,9,10,11].

The nanodrug delivery platform is a subject of rapid growth because of its significant role in the improvement of therapeutic values of different drugs and bioactive molecules by modulating their release profiles, bioavailability, and solubility, and protecting the drug from degradation [5,12,13]. Increased efforts are now being invested into engineering multifunctional, biocompatible nanomaterials for the delivery of drugs at the site of infection thereby resulting in more effective therapy [14,15,16,17]. Different nanomaterials such as liposomes, polymers, micelles, carbon nanotubes, quantum dots, dendrimers, metallic nanoparticles, and graphene oxide (GO) have been applied for the delivery of different drugs and biomolecules [18,19,20,21,22,23,24,25,26,27].

Among all of these nanomaterials, GO has garnered the most attention due to its favourable electrical, chemical, thermal, optical, mechanical, and biological properties. GO is cheap, has good dispersibility in water and physiological solutions, good tensile strength, the sustained-release property, large surface area, low toxicity, and excellent biocompatibility making it an ideal candidate for drug delivery applications [12,28,29,30]. The 2D aromatic surface with oxygenated functional groups of GO makes it an ideal substrate for drug loading via different electrostatic interactions between the drugs and the host, GO [12,30].

Ethambutol [(+)-2,2-(ethylenediimino)di-1-butanol)] (ETB) has been used for the treatment of tuberculosis since 1966 [31]. The initial dose of ethambutol is 15 mg/kg of body weight per day. It is a first line anti-TB drug with side effects such as loss of appetite, headache, nausea, stomach upset, dizziness, vomiting, abdominal pain, worsening gout, or joint pain [31,32]. In this study we have designed an anti-tuberculosis nanodelivery formulation using GO as the nanocarrier and ETB as the guest drug. The designed formulation was thoroughly characterized using XRD, FTIR, TEM, HPLC and Raman spectroscopy. The designed formulation was also evaluated for in vitro sustained release, biocompatibility and therapeutic efficacy.

## 2. Results

### 2.1. Physico-Chemical Characterisation of ETB-GO

#### 2.1.1. Powder X-ray Diffraction (XRD)

**Figure 1:**
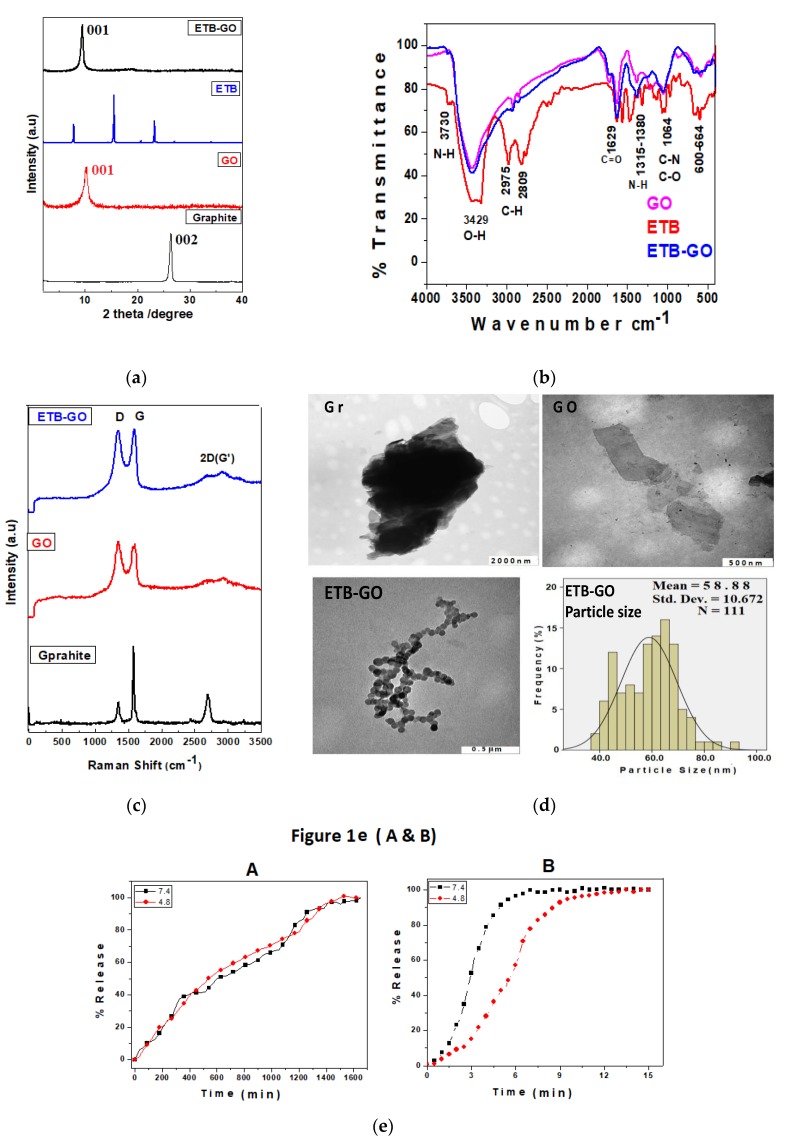
(**a**) XRD patterns of graphite (Gr), Graphene oxide (GO), ETBambutol (ETB), and the nanodelivery formulation ETB-GO. (**b**) FTIR spectra of the nanocarrier GO, free drug ETB, and ETB-GO; (**c**) Raman spectra of Gr, GO, and ETB-GO. (**d**) Transmission electron micrographs of Gr, GO, and ETB-GO, and the particle size distribution of ETB-GO. (**e**) (**A**) In vitro release of ETB from the nanodelivery formulation ETB-GO in PBS solution of pH 7.4 and PBS solution of pH 4.8. In vitro release profile of free drug ETB in PBS solution of pH 7.4 and pH 4.8 (**e**) (**B**).

Figure 1a shows the XRD patterns of graphite (Gr), the nanocarrier graphene oxide (GO), the free drug ethambutol and the nanodelivery formulation composed of ethambutol loaded on graphene oxide (ETB-GO). The starting material Gr showed a characteristic sharp peak due to the diffraction pattern of the highly graphitic plane (002) at 2θ = 26.27°, having well-organized layers with basal spacing of 3.4 Å [5,12]. The nanocarrier GO was prepared from Gr, and the successful formation of GO is confirmed by the disappearance of the graphite peak at 2θ = 26.27° and the appearance of a new peak of GO at about 2θ = 10.0°, attributed to the diffraction of the (001) planes of GO with increased basal spacing of 8.8 Å [33]. The increase in the basal spacing of GO can be attributed to the increase in the interlayer distance, exfoliation of Gr layers, and formation of oxygenated layers of GO [15]. Three crystalline intense peaks were observed for the pure drug ETB at 2θ of 7.86°, 15.48° and 23.25°, corresponding to the crystalline characteristics of the organic molecule [12]. In contrast, the nanodelivery formulation ETB-GO showed a small hub of ETB peaks at 15.48°, which shows amorphous characteristics compared with the XRD pattern of the pure drug. The absence of the XRD pattern of the organic drug molecules after loading is a normal phenomenon and has been reported previously [5,12,34]. However, the GO peak is present in the nanocomposite formulation, ETB-GO. The successful loading of ETB was further confirmed by other complementary techniques and will be discussed later.

#### 2.1.2. Fourier Transformed Infrared Analysis

Fourier transformed infrared (FTIR) analysis is the major analytical technique used for the detection of functional groups of molecules. Figure 1b shows the FTIR spectra of ETB, GO, and ETB-GO. The FTIR spectrum of GO shows the characteristic bands of oxygenated functional groups such as a hydroxyl stretching absorption bands at 3429 cm^−1^, a carbonyl (C=O) stretching band at 1629 cm^−1^, and alkoxyl and epoxide stretching bands at about 1064 cm^−1^ [5,12,33,35]. The free drug showed characteristic functional group bands of N–H stretching at about 3739 cm^−1^, O–H stretching vibration at about 3429 cm^−1^, C–H stretching vibration at 2975 cm^−1^ and 2809 cm^−1^, and a C–N stretching vibration band at about 1060 cm^−1^ [36,37]. The FTIR spectrum of the nanodelivery formulation ETB-GO showed the FTIR vibrational bands of both the nanocarrier and the pure ETB at similar positions. The presence of functional group bands belonging to ETB and GO in the spectrum of the nanodelivery formulation is strong evidence for the successful formation of the ETB-GO.

#### 2.1.3. Raman Spectroscopy

Raman spectroscopy is a powerful tool to analyze the disorder, defects, and structural changes in carbon-based material after chemical modifications [38]. Figure 1c shows the Raman spectra of Gr, GO and ETB-GO. The Raman spectrum of Gr shows two main graphitic characteristic peaks: the D–band corresponding to the disorder-induced mode and the G-band ascribed to the graphitic-like mode appeared at 1349 cm^−1^ and 1557 cm^−1^, respectively [38]. The D-band, corresponding to the nanocrystalline quality of the carbon structures, is due to disorder or defects in the material and its high intensity is due to the presence of more sp^2^ domains. The G-band is related to the high degree of order of the crystalline graphitic structure [39,40,41]. The successful formation of the nanocarrier GO was confirmed by the shift and the intensity change of the D and G bands at 1249 cm^−1^ and the G band at 1594 cm^−1^, respectively. The nanodelivery formulation also showed the shift in D and G bands to 1357 cm^−1^ and 1581 cm^−1^ respectively. The I_D_/I_G_ ratio for GO and ETB-GO was found to be 1.0 compared to 0.833 for Gr. The increase in value of the I_D_/I_G_ ratio is in agreement with previous studies [5,39]. The results from the Raman studies strongly complement the XRD and FTIR results, which support the successful formation of the nanocarrier GO and the nanocomposite formulation ETB-GO.

#### 2.1.4. Transmission Electron Microscopy (TEM) Analysis

The structural features and morphology of the starting material (graphite), the nanocarrier, and the nanodelivery formulation were determined using transmission electron microscopy. Figure 1d shows the TEM micrographs of the three materials together with the particle size distribution of ETB-GO. It is very clear from TEM micrograph of Gr that it contains a bunch of graphene sheets stacked together. However, we can observe a single sheet of GO in the TEM micrograph, which is strong evidence for the successful oxidative exfoliation of Gr to GO. The TEM micrograph of the nanodelivery formulation ETB-GO shows the particulate formation after the loading process. The particle size of ETB-GO was determined using Image analysis software and SPSS software. About 111 particles (N) were randomly selected and their mean size was determined to be 59 nm with a standard deviation of 11 nm.

#### 2.1.5. In Vitro Release Study

Ultraviolet-visible (UV/Vis) spectroscopy was used for the in vitro release of ETB from ETB-GO and for the release profile of the free drug ETB in human body-simulated phosphate buffered saline (PBS) solutions of pH 7.4 and pH 4.8. Figure 1e (A) shows the in vitro release behavior of ETB from ETB-GO in PBS solutions of pH 7.4 and pH 4.8. The release was found to be similar at both pH levels tested. The sustained release took about 1500 min (25 h) for the solution to become saturated and a plateau to be observed, which suggests that the release was completed by that time period. The mechanism of release of the drug from the GO nanocarrier has been reported to involve the ion exchange process of GO with the anions present in the buffer solutions [5,41,42,43]. The sustained release of ETB up to 1500 min also suggests the successful loading of the ETB on the GO with a strong interaction between them. The free drug ETB took about 10 min for complete release as shown in Figure 1e (B), which is far faster in comparison with the release from the designed formulation. The sustained release would enhance the bioavailability of the drug which would translate to improving the therapeutic efficacy and therefore may also allow for the reduction in dosing frequency of the drug.

### 2.2. Biological Evaluation of ETB-GO

#### 2.2.1. Antimycobacterial Activity of ETB-GO on Planktonic Cells

**Figure 2:**
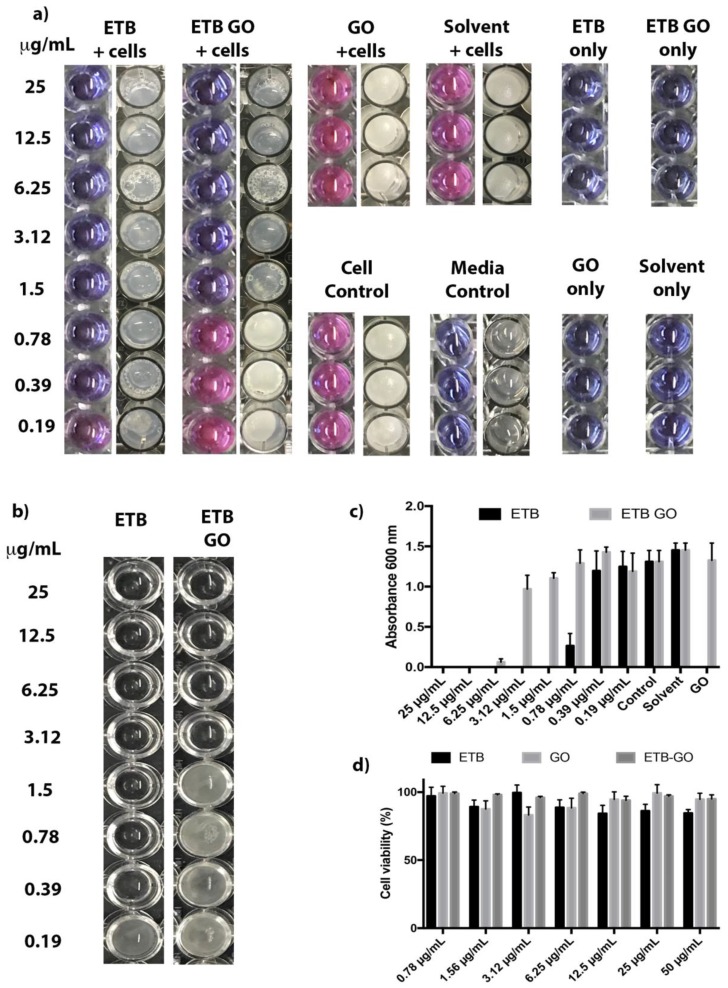
(**a**) REMA and SPOTi results based on ethambutol (ETB) and the multifunctional nanoformulation ETB-GO tested on *M. smegmatis*. (**b**) *M. smegmatis* biofilm treated with ETB and ETB-GO. The MIC of ETB-GO was found to be 1.5 μg/mL, however, when treating biofilm, the MIC was found 1 fold higher (3.12 μg/mL). (**c**) Quantification of concentration-dependent biofilm of ETB and ETB-GO by crystal violet. (**d**) Cell viability of 3T3 cells after 72 h incubation with ETB, GO, and ETB-GO.

Figure 2a shows the growth inhibition properties of ETB, GO, and ETB-GO on *M. smegmatis.* The MIC of the free drug ETB and of the nanodelivery formulation was found to be 0.39 μg/mL and 1.5 μg/mL, respectively, as shown in Table 1. However, the percentage loading of ETB in the nanodelivery formulation was found to be 47.96%, determined by using HPLC. Based on this, the amount of ETB present in 1.5 μg of ETB-GO would be 0.72 μg (effective MIC). A similar anti-mycobacterial effect is also observed in the modified SPOTi assay (Table 1). This indicates drug loading and release processes do not considerably affect the activity of the drug ETB.

#### 2.2.2. Biofilm Inhibition by ETB-GO

ETB-GO was found to inhibit *M. smegmatis* biofilm formation at 3.12 μg/mL (effective concentration 1.496 µg/mL) which is higher than the MIC of the planktonic bacterial cells (Figure 2b and c). This is not unusual as biofilms are tolerant of external pressures such as antibiotics and require higher exposures for longer periods of time for their elimination [44]. ETB biofilm inhibitory properties have been reported earlier [45]. Overall, ETB was better in inhibiting the *M. smegmatis* biofilm than the nanoformulation. The reduced activity may indicate that the slow release of the drug is not effective against a biofilm colony.

#### 2.2.3. Cytotoxicity Studies

The biocompatibility of the material considered for health care applications is the key aspect of drug delivery research. These studies were performed on 3T3 mouse fibroblast cells wherein the cells were incubated for maximum incubation period of 72 h with ETB, GO, and ETB-GO using a concentration gradient between 0.78 μg/mL and 50 μg/mL. The cell viability was determined using MTT assay. After 72 h of incubation, all of the samples of ETB, GO, and ETB-GO were found to be highly biocompatible with more than 85% cell viability as shown in Figure 2d. The nanodelivery formulation ETB-GO was found to be more biocompatible (cell viability more than 95%) compared to the free drug ETB (cell viability about 85%) at the highest concentration 50 μg/mL. This suggests that the designed nanodelivery formulation ETB-GO and its starting material, GO, are highly biocompatible and are safe for health care applications. The cellular uptake mechanism of the GO-based nanoformulation is reported to be via endocytosis; uptake begins immediately and smaller nanoparticles with higher concentration are taken up 1000 times higher than large GO flakes [46,47]. The graphene-based nanoformulation is removed by the phagocytosis process [48].

## 3. Discussion

In this study we have designed an anti-TB nanodelivery formulation using graphene oxide as the nanocarrier and an anti-TB drug (ethambutol) as the guest drug. The successful formation of a nanodelivery formulation was confirmed by various detailed instrumental characterizations. The designed ETB-GO was found to release the drug in sustained manner over an extended period of 25 h in human body-simulated PBS solutions of pH 7.4 and pH 4.8. Sustained-release formulations provide controlled dispersal of the drug, thereby maximizing the time during which the peak concentration is maintained in a patient while minimizing the cytotoxic effects [49]. Augmentin XR (amoxicillin/clavulanate with hypromellose) is a slow-release antibiotic formulation currently on the market which has improved treatment outcomes and reduced treatment costs [50]. Zhu et al. reported lower than expected maximum serum concentrations (C_max_) in adults [51]. In pediatric patients this value was lower and delayed absorption of the drug was also reported. Many C_max_ were found to be sub-minimal inhibitory concentrations, allowing for the development of a resistant-strain selective environment. Nanoformulations and alternate drug delivery systems or administration routes may impact the pharmacokinetics of the drug to the benefit of the patient by improving absorption and retention periods in systemic circulation.

Graphene oxide has achieved much success as a drug delivery system for anti-cancer drugs since the first report by Liu et al. [52]. The ethambutol-containing nanodelivery formulation was found to be biocompatible with 95% of the 3T3 cells surviving an exposure of 50 μg/mL of the nanoformulation over a period of 72 h. The therapeutic efficacy determined using REMA, modified SPOTi, and biofilm inhibition against *Mycobacterial smegmatis* revealed that ETB retains its growth inhibitory property.

Sustained release of the potent first-line anti-TB drug would be beneficial for improving the drug bioavailability and therapeutic efficacy with reduced dosing frequency. This modification would have a significant impact on the design of drug treatment regimens with implications of improving patient compliance. Therefore we believe these results provide a firm basis for in vivo studies.

## 4. Materials and Methods

### 4.1. Chemicals

Ethambutol dihydroxychloride (99% purity), potassium permanganate (KMnO_4_), hydrogen peroxide (H_2_O_2_), graphite flakes with 109 meshes, sulphuric acid (H_2_SO_4_, 98%), phosphate-buffered saline, and phosphoric acid (H_3_PO_4_) were purchased from Sigma Aldrich (St Louis, MO, USA) and used without further purification. Diethyl ether, sodium hydroxide, and hydrochloric acid (HCl, 37%) were purchased from Friedemann Schmidt (Parkwood, WA, USA). Normal fibroblast (3T3) cell lines were purchased from the American Tissue Culture Collection (Manassas, VA, USA) and deionized water was used in all experiments.

### 4.2. Instrumentation

For X-ray diffraction (XRD) studies, a Shimadzu XRD-6000 Diffractometer (Shimadzu Corporation, Tokyo, Japan), was utilized with CuK_α_ radiation at 30 kV and 30 mA. For the functional group analysis, a Perkin-Elmer (Waltham, MA, USA) 100 series spectrophotometer Fourier Transform infrared (FTIR) was used, and spectra were recorded by the direct sample method. For the optical and release studies, a Lambda 35 ultraviolet-visible spectrophotometer (Perkin Elmer) was used. A Raman spectrometer, Witec Model: Alpha 300R was used for the RAMAN analysis of the samples with 532 nm excitation. For drug quantification, Sykam high performance liquid chromatography (HPLC) system (SYKAM GmbH, Eresing, Germany) was used with the auto injector Sykam 5300, a Sykam S3250 UV/Vis detector, and the Sykam quaternary pump system 5300 made in Germany, with a Zorbax Rx-Sil column of 4.6 × 150 mm, and with 5 μm particle size (Agilent Technologies, Santa Clara, CA, USA).

### 4.3. Synthesis of Graphene Oxide

The graphene oxide was synthesized by oxidative exfoliation using improved Hummer’s method. In brief, 3 g of graphite powder and 18 g of KMnO_4_ were added to 400 mL (360 mL concentrated H_2_SO_4_ + 40 mL concentrated H_3_PO_4_) and the solution was then stirred continuously at a constant heat of 50 °C for 12 h. After 12 h, the sample was allowed to cool and then poured on 400 g of ice cubes containing 3 mL of H_2_O_2_. The final product was subjected to work-up by washing with deionized water, concentrated HCl, and ethanol (200 mL of each, respectively), and then coagulated with 200 mL diethyl ether. The product was dried in an oven at 40 °C [33].

### 4.4. Preparation of Anti-TB Nanodelivery Formulation (ETB-GO)

The anti-TB nanodelivery formulation was prepared by loading the anti-TB drug ETB onto graphene oxide (ETB-GO), by adding 0.2 g of GO into 2% solution of ETB and maintaining the pH of the mixture at pH = 5. The sample was stirred overnight at room temperature and the resulting slurry was washed thoroughly with water, centrifuged, and dried at 40 °C.

### 4.5. In Vitro Sustained Release

Release studies were conducted in human body-simulated phosphate buffer saline (PBS) solution of 0.1 M with pH of 7.4 (blood pH) and 4.8 (intracellular lysosomal pH). About 4 mg of the nanodelivery formulation of ETB-GO was added into 3.5 mL of pH 7.4 and 4.8 solutions separately and the absorption was taken at 190 nm in a UV/Vis spectrophotometer for the release profile of ETB.

### 4.6. Drug Loading Quantification by HPLC Analysis

The loading of ETB was quantified using HPLC analysis by utilizing the previously reported method with a slight modification [1]. In brief, the mobile phase comprised solvent A (acetonitrile) and solvent B (15 mmol/L potassium dihydrogen phosphate buffer solution), with pH adjusted to 4.0 ± 0.1, used in a ratio of 89:11 with a flow rate of 1 mL/min at 25 °C. A UV detector was used with the selected wavelength of 192 nm. Different concentrations of ETB standard solution were used for a calibration curve determination and R^2^ was found to be 0.9973. The retention time for ETB elution was found to be 2.3 min and the % loading was determined to be 47.96%.

### 4.7. In Vitro Cytotoxicity Study

#### MTT Assay for In Vitro Cytotoxicity Studies

For the evaluation of the cytotoxicity profiles, MTT [3-(4,5-dimethylthiazol-2-yl)-2,5-diphenyltetrazolium bromide] assay was used as reported previously [1,53,54]. In brief, the eukaryotic cells (3T3) were seeded into a 96-well plate with the density of 1.0 × 10^5^ cells per well in 100 μL of cell culture medium, and allowed to attach for 24 h before addition of the samples. After 24 h, all the samples ETB, GO, and ETB-GO were individually added to the cells at various concentrations (0.78 to 50 μg/mL), and incubated for 72 h at 37 °C in a humidified chamber (5% CO_2_ atmosphere). The untreated 3T3 fibroblast cells (cells only, free from any drug or nanoformulation) were considered as an experimental control. Samples were treated in triplicate for each concentration. Following the 72 h incubation period, 20 μL of MTT solution (with a concentration of 5 mg/mL) was added to each well and incubated at 37 °C for 4 h. Four hours after MTT addition, dimethyl sulfoxide (100 μL/well) was added to each well to dissolve the dark blue formazan crystals formed due to the reduction of tetrazolium by living cells. The concentration of living cells was determined by measuring the concentration of blue formazan at 570 nm and 630 nm using a microplate enzyme-linked immunosorbent assay reader (ELx800, BioTek Instruments, Winooski, VT, USA) [53,54]. Cell viability data were stated as a percentage by comparing with the untreated cells under identical experimental conditions. All experiments were carried out in triplicate, and the results are presented as the mean ± standard deviation.

### 4.8. Antimycobacterial Assays

#### 4.8.1. Resazurin Microtiter Plate Assay (REMA)

The minimum inhibitory concentration (MIC) of the drug and its nanoformulation were established by Resazurin Microtiter Plate Assay (REMA). In brief, 2 µL of the drug/nanoformulation stock of concentration 5 mg/mL was added to 198 µL of media in a microtiter plate and serially diluted along the columns of the plate. *M. smegmatis* cells were grown until early-logarithmic phase (OD_600_ ~1.00), then diluted (10^−3^ dilution), and 100 µL of the cell suspension was added to each well. Solvent (DMSO), carrier (GO), drug-free, and cell-free controls were set up. The plates were incubated for 24 h at 37 °C. After incubation, 30 µL of resazurin dye mixture (1:1 (*v*/*v*) of 0.01% (*w*/*v*) resazurin and 20% (*v*/*v*) Tween-80%) was added to each well and incubated for a further 6–9 h. A change in colour of the solution from blue to pink indicates oxidation brought about by active growth of bacteria. The MIC was determined by the lowest concentration of the compound wherein no colour change is observed. The experiment was performed in triplicate.

#### 4.8.2. Modified SPOTi Assay

This assay is modified based on the SPOTi experimental method [55,56,57,58] to evaluate formulated compounds more appropriately. The microtiter plate with the drug/nanoformulation and the *M. smegmatis* cells were prepared as for the REMA. Solvent (DMSO), carrier (GO), drug-free, and cell-free controls were set up. The tubes were incubated for 24 h at 37 °C shaking at 150 rpm to keep the nanoformulations uniformly dispersed. After completion of incubation, 2 µL of the cell suspension was spotted onto microtiter plate with each well containing 200 µL of M7H10 media (BD Biosciences, Wokingham Berkshire, England) supplemented with 0.02% (*v*/*v*) glycerol and 10% Oleic acid-Albumin-Dextrose-Catalase (OADC, BD Biosciences, UK). The plate was incubated at 37 °C for 48 h or until visible spots of bacterial growth appeared in the drug-free controls. The MIC was determined as the lowest concentration of the drug/nanoformulation at which no growth of bacteria was observed. The experiment was performed in triplicate.

#### 4.8.3. Biofilm Inhibition

Biofilm growth and inhibition assay was set up in 96-well plates (Corning, polystyrene, non-treated). 10 µL of late-logarithmic phase (OD_600_ ~4) *M. smegmatis* cells were added to each well and the final volume made up to 200 µL with Sauton’s media (0.5 g KH_2_PO_4_, 0.5 g MgSO_4_, 4.0 g L-asparagine, 0.05 g Ferric ammonium citrate, 2.0 g citric acid, and 60 mL glycerol dissolved in 900 mL dH_2_O and pH adjusted to 7) containing the drug and its formulation in a range of concentrations between 25 µg/mL and 0.19 µg/mL. Solvent (DMSO), carrier (GO), drug-free, and cell-free controls were set up. The biofilms were allowed to grow for 5 days at 37 °C under static conditions. At the end of the incubation period, the media containing planktonic cells was aspirated and the biofilm was dried in an oven at 50 °C for 10 min. A quantity of 200 µL of crystal violet solution (0.1%) was added to each well and left to incubate for 30 min at room temperature. Finally, 30% acetic acid was added to solubilize the biofilm, followed by 30 min of incubation at room temperature. The contents of each well was transferred to a fresh 96-well plate and the absorbance at 600 nm recorded using a Biotek (Synergy) plate reader. The experiment was performed in triplicate.

## Figures and Tables

**Table 1:** The minimum inhibitory concentration of the compounds tested based on the liquid culture REMA assay with *M. smegmatis*.

Compound	REMA		Modified SPOTi	
	Observed MIC (µg/mL)	Effective MIC (µg/mL)	Observed MIC (µg/mL)	Effective MIC (µg/mL)
ETB	0.39	0.39	0.39	0.39
ETBGO	1.5	0.72	1.5	0.72

## References

[1] Saifullah B., El Zowalaty M.E., Arulselvan P., Fakurazi S., Webster T.J., Geilich B.M., Hussein1 M.Z. (2016). Synthesis, characterization, and efficacy of antituberculosis isoniazid zinc aluminum-layered double hydroxide based nanocomposites. Int. J. Nanomed..

[2] Saifullah B., Hussein M.Z., Hussein Al Ali S.H. (2012). Controlled-release approaches towards the chemotherapy of tuberculosis. Int. J. Nanomed..

[3] World Health Organization (WHO) (2016). Global Tuberculosis Report.

[4] Ellingsen L.A.-W., Hung C.R., Majeau-Bettez G., Singh B., Chen Z., Whittingham M.S., Stromman A.H. (2017). Corrigendum: Nanotechnology for environmentally sustainable electromobility. Nat. NanoNanotechnol..

[5] Barahuie F., Saifullah B., Dorniani D., Fakurazi S., Karthivashan G., Hussein M.Z., Elfghi F.M. (2017). Graphene oxide as a nanocarrier for controlled release and targeted delivery of an anticancer active agent, chlorogenic acid. Mater. Sci. Eng. C.

[6] Lu J., Chen Z., Ma Z., Pan F., Curtiss L.A., Amine K. (2016). The role of nanotechnology in the development of battery materials for electric vehicles. Nat. NanoNanotechnol..

[7] Moghimi S.M., Hunter A., Murray J.C. (2005). Nanomedicine: Current status and future prospects. FASEB J..

[8] Yang N., Jiang X. (2017). Nanocarbons for DNA sequencing: A review. Carbon.

[9] Zhang Z.-Y., Guo W. (2017). Cutting monolayer graphene into flexible spin filters. Carbon.

[10] Ray S.C. (2015). Chapter 1—Application and Uses of Graphene. Applications of Graphene and Graphene—Oxide Based Nanomaterials.

[11] Xia F., Wang H., Xiao D., Dubey M., Ramasubramaniam A. (2014). Two-dimensional material nanophotonics. Nat. Photon..

[12] Dorniani D., Saifullah B., Barahuie F., Arulselvan P., Hussein M.Z.B., Fakurazi S., Twyman L.J. (2016). Graphene oxide-gallic acid nanodelivery system for cancer therapy. Nanoscale Res. Lett..

[13] Posati T., Bellezza F., Tarpani L., Perni S., Latterini L., Marsili V., Cipiciani A. (2012). Selective internalization of ZnAl-HTlc nanoparticles in normal and tumor cells. A study of their potential use in cellular delivery. Appl. Clay Sci..

[14] Tinkle S., McNeil S.E., Mühlebach S., Bawa R., Borchard G., Barenholz Y., Tamarkin L., Desai N. (2014). Nanomedicines: Addressing the scientific and regulatory gap. Ann. N.Y. Acad. Sci..

[15] Taghdisi S.M., Danesh N.M., Lavaee P., Emrani A.S., Hassanabad K.Y., Ramezani M., Abnous K. (2016). Double targeting, controlled release and reversible delivery of daunorubicin to cancer cells by polyvalent aptamers-modified gold nanoparticles. Mater. Sci. Eng. C.

[16] Wagner V., Dullaart A., Bock A.-K., Zweck A. (2006). The emerging nanomedicine landscape. Nat. Biotech..

[17] Alexis F., Rhee J.-W., Richie J.P., Radovic-Moreno A.F., Langer R., Farokhzad O.C. (2008). New frontiers in nanotechnology for cancer treatment. Urol. Oncol. Semin. Orig. Investig..

[18] Masood F. (2016). Polymeric nanoparticles for targeted drug delivery system for cancer therapy. Mater. Sci. Eng. C.

[19] Elgadir M.A., Uddin M.S., Ferdosh S., Adam A., Chowdhury A.J.K., Sarker M.Z.I. (2015). Impact of chitosan composites and chitosan nanoparticle composites on various drug delivery systems: A review. J. Food Drug Anal..

[20] Pattni B.S., Chupin V.V., Torchilin V.P. (2015). New developments in liposomal drug delivery. Chem. Rev..

[21] Yong K.-T., Wang Y., Roy I., Rui H., Swihart M.T., Law W.-C., Kwak S.K., Ye L., Liu J., Mahajan S.D. (2012). Preparation of quantum dot/drug nanoparticle formulations for traceable targeted delivery and therapy. Theranostics.

[22] Martincic M., Tobias G. (2015). Filled carbon nanotubes in biomedical imaging and drug delivery. Expert Opin. Drug Deliv..

[23] Kesharwani P., Jain K., Jain N.K. (2014). Dendrimer as nanocarrier for drug delivery. Prog. Polym. Sci..

[24] Yan T., Zhang H., Huang D., Feng S., Fujita M., Gao X.-D. (2017). Chitosan—Functionalized graphene oxide as a potential immunoadjuvant. Nanomaterials.

[25] Xu L., Wan C., Du J., Li H., Liu X., Yang H., Li F. (2017). Synthesis, characterization, and in vitro evaluation of targeted gold nanoshelled poly(d,l-lactide-CO-glycolide) nanoparticles carrying anti p53 antibody as a theranostic agent for ultrasound contrast imaging and photothermal therapy. J. Biomater. Sci. Polym. Ed..

[26] Shi J., Kantoff P.W., Wooster R., Farokhzad O.C. (2017). Cancer nanomedicine: Progress, challenges and opportunities. Nat. Rev. Cancer.

[27] Liu J., Cui L., Losic D. (2013). Graphene and graphene oxide as new nanocarriers for drug delivery applications. Acta Biomater..

[28] Feng L., Liu Z. (2011). Graphene in biomedicine: Opportunities and challenges. Nanomedicine.

[29] Sun X., Liu Z., Welsher K., Robinson J.T., Goodwin A., Zaric S., Dai H. (2008). Nano-graphene oxide for cellular imaging and drug delivery. Nano Res..

[30] Sahu A., Choi W.I., Lee J.H., Tae G. (2013). Graphene oxide mediated delivery of methylene blue for combined photodynamic and photothermal therapy. Biomaterials.

[31] (2008). Ethambutol. Tuberculosis.

[32] Prasad R., Garg R., Verma S.K. (2008). Isoniazid- and ethambutol-induced psychosis. Ann. Thorac. Med..

[33] Marcano D.C., Kosynkin D.V., Berlin J.M., Sinitskii A., Sun Z., Slesarev A., Alemany L.B., Lu W., Tour J.M. (2010). Improved synthesis of graphene oxide. ACS Nano.

[34] Barahuie F., Hussein M.Z., Arulselvan P., Fakurazi S., Zainal Z. (2016). Controlled in vitro release of the anticancer drug chlorogenic acid using magnesium/aluminium-layered double hydroxide as a nanomatrix. Sci. Adv. Mater..

[35] Verma S., Mungse H.P., Kumar N., Choudhary S., Jain S.L., Sain B., Khatri O.P. (2011). Graphene oxide: An efficient and reusable carbocatalyst for Aza-Michael addition of amines to activated alkenes. Chem. Commun..

[36] Ahmad M.I., Ungphaiboon S., Srichana T. (2015). The development of dimple-shaped chitosan carrier for ethambutol dihydrochloride dry powder inhaler. Drug Dev. Ind. Pharm..

[37] Annapurna M.M., Rao M.E.B., Kumar B.V.V.R. (2006). Synthesis, Spectral characterization and evaluation of pharmacodynamic activity of copper and nickel complexes of ethambutol dihydrochloride. E-J. Chem..

[38] King A.A.K., Davies B.R., Noorbehesht N., Newman P., Church T.L., Harris A.T., Razal J.M., Minett A.I. (2016). A new raman metric for the characterisation of graphene oxide and its derivatives. Sci. Rep..

[39] Gurunathan S., Han J.W., Kim E.S., Park J.H., Kim J. (2015). Reduction of graphene oxide by resveratrol: A novel and simple biological method for the synthesis of an effective anticancer nanotherapeutic molecule. Int. J. Nanomed..

[40] Kalbac M., Hsieh Y.-P., Farhat H., Kavan L., Hofmann M., Kong J., Dresselhaus M.S. (2010). Defects in individual semiconducting single wall carbon nanotubes: Raman spectroscopic and in situ raman spectroelectrochemical study. Nano Lett..

[41] Dresselhaus M.S., Jorio A., Hofmann M., Dresselhaus G., Saito R. (2010). Perspectives on carbon nanotubes and graphene raman spectroscopy. Nano Lett..

[42] Lv Y., Tao L., Annie Bligh S.W., Yang H., Pan Q., Zhu L. (2016). Targeted delivery and controlled release of doxorubicin into cancer cells using a multifunctional graphene oxide. Mater. Sci. Eng. C Mater. Biol. Appl..

[43] Wang H., Gu W., Xiao N., Ye L., Xu Q. (2014). Chlorotoxin-conjugated graphene oxide for targeted delivery of an anticancer drug. Int. J. Nanomed..

[44] Pratten J., Ready D. (2010). Use of biofilm model systems to study antimicrobial susceptibility. Antibiotic Resistance Protocols.

[45] Sachan T.K., Kumar V. (2015). Antibiotic susceptibility in biofilms of *Mycobacterium smegmatis*. Int. J. Appl. Sci. Biotechnol..

[46] Mendes R.G., Mandarino A., Koch B., Meyer A.K., Bachmatiuk A., Hirsch C., Schmidt O.G., Gemming T., Liu Z., Rümmeli M.H. (2017). Size and time dependent internalization of label-free nano-graphene oxide in human macrophages. Nano Res..

[47] Wu S.Y., An S.S.A., Hulme J. (2015). Current applications of graphene oxide in nanomedicine. Int. J. Nanomed..

[48] Mukherjee S.P., Bottini M., Fadeel B. (2017). Graphene and the immune system: A romance of many dimensions. Front. Immunol..

[49] Gao P., Nie X., Zou M., Shi Y., Cheng G. (2011). Recent advances in materials for extended-release antibiotic delivery system. J. Antibiot..

[50] Jackson J., Fernandes A.W., Nelson W. (2006). A naturalistic comparison of amoxicillin/clavulanate extended release versus immediate release in the treatment of acute bacterial sinusitis in adults: A retrospective data analysis. Clin. Ther..

[51] Zhu M., Burman W., Starke J., Stambaugh J., Steiner P., Bulpitt A., Ashkin D., Auclair B., Berning S., Jelliffe R. (2004). Pharmacokinetics of ethambutol in children and adults with tuberculosis. Int. J. Tuberc. Lung Dis..

[52] Liu Z., Robinson J.T., Sun X., Dai H. (2008). PEGylated nanographene oxide for delivery of water-insoluble cancer drugs. J. Am. Chem. Soc..

[53] Chen H., Pu J., Liu D., Yu W., Shao Y., Yang G., Xiang Z., He N. (2016). Anti-inflammatory and antinociceptive properties of flavonoids from the fruits of black mulberry (*Morus nigra* L.). PLoS ONE.

[54] Ahmadian S., Barar J., Saei A.A., Fakhree M.A.A., Omidi Y. (2009). Cellular toxicity of nanogenomedicine in MCF-7 cell line: MTT assay. J. Vis. Exp..

[55] Danquah C.A., Maitra A., Gibbons S., Faull J., Bhakta S. (2016). HT-SPOTi: A rapid drug susceptibility test (DST) to evaluate antibiotic resistance profiles and novel chemicals for anti-infective drug discovery. Curr. Protoc. Microbiol..

[56] Rizi K., Murdan S., Danquah C.A., Faull J., Bhakta S. (2015). Development of a rapid, reliable and quantitative method—“SPOTi” for testing antifungal efficacy. J. Microbiol. Methods.

[57] Guzman J.D., Evangelopoulos D., Gupta A., Birchall K., Mwaigwisya S., Saxty B., McHugh T.D., Gibbons S., Malkinson J., Bhakta S. (2013). Antitubercular specific activity of ibuprofen and the other 2-arylpropanoic acids using the HT-SPOTi whole-cell phenotypic assay. BMJ Open.

[58] Evangelopoulos D., Bhakta S. (2010). Rapid methods for testing inhibitors of mycobacterial growth. Antibiotic Resistance Protocols.

